# Pleural metastasis of anaplastic meningioma

**DOI:** 10.1016/j.radcr.2020.10.020

**Published:** 2020-10-19

**Authors:** G.D. Marijn Veerman, Martin J. van den Bent, Marthe S. Paats

**Affiliations:** a Department of Medical Oncology, Erasmus MC Cancer Institute, Erasmus University Medical Centre, PO box 2040, 3000 CB, Rotterdam, The Netherlands; b Department of Neurology, Erasmus MC, Rotterdam, The Netherlands; c Department of Pulmonology, Erasmus MC, Rotterdam, The Netherlands

**Keywords:** Anaplastic meningioma, Pleural metastasis

## Abstract

A 52-year-old woman presented to the emergency department with several days of progressive dyspnoea and thoracic pain. Her medical history included a (recurrent) anaplastic meningioma, for which she was treated with surgery and radiotherapy. A chest X-ray showed occurrence of total opacification of the left lower lobe and a chest computed tomography demonstrated a pleural mass of 12 × 9 × 15 cm in the left lower lobe. Biopsy of the pleural mass revealed a metastasis of the patient's anaplastic meningioma. Extracranial metastases from meningioma are extremely uncommon (≤ 0.1%-0.2% of cases), but important for a patient's prognosis.

## Introduction

Meningioma is with 30% the most frequent occurring malignancy of the central nervous system, and has an incidence rate of 4.5 cases in 100,000 people each year. Meningiomas originate from the meninges and are typically associated with genetic alterations in the neurofibromatosis type 2 gene. Diagnosis is based on a magnetic resonance imaging (MRI) scan and confirmed by histopathological analysis of the tumor [1]. Meningiomas are graded by the World Health Organisation (WHO) classification system in 3 subtypes [2]. In approximately 80% of cases the meningioma is benign (WHO grade 1) [3]. The surplus consists of atypical meningioma (WHO grade 2; 15% to 20%) and anaplastic -or malignant-meningioma (WHO grade 3; 1% to 3%) [1,4].

First-line treatment of benign meningioma is largely dependent on patient specific characteristics (eg, age and performance status) and patient preference. In higher grade meningioma however, first-line treatment is preferably surgical resection, and in case of grade 3 meningioma followed by high-dose radiotherapy, in order to minimize the change of local disease recurrence [5]. Nevertheless, in 50%-90% of anaplastic meningiomas the tumor recurs [3,6]. Hence frequent follow-up by MRI is additionally recommended [5]. When anaplastic meningioma does locally recur or metastasize to other locations, treatment options are limited. Therefore, the median overall survival of anaplastic meningioma is less than 2 years [7]. At reoccurrence, surgical resection or reirradiation of the tumor(s) could be possible, but clinical efficacy data are solely from case reports. Current guidelines do not (yet) consider pharmacotherapy to have a place in the treatment of recurrent or metastasized anaplastic meningioma [3,5].

This case report describes the discovery of a rare pleural metastasis in a patient with recurrent anaplastic meningioma.

## Case report

**Fig. 1:**
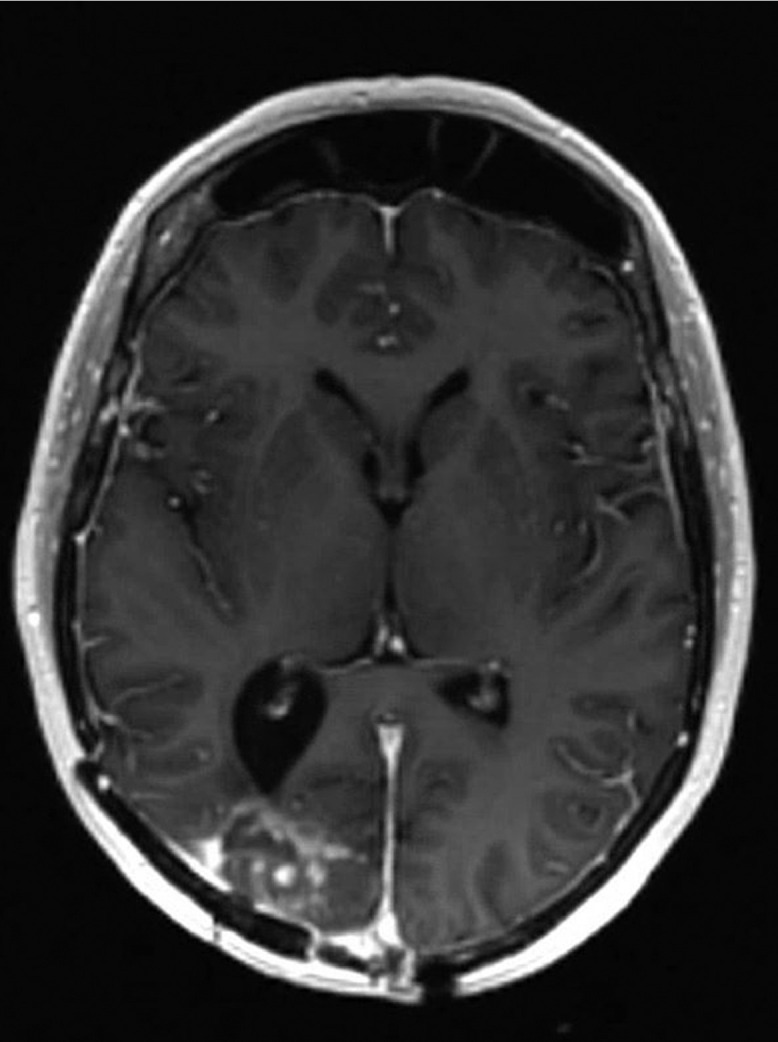
Magnetic resonance image of the brain in April 2019; local recurrence predominantly in the right occipital lobe.

**Fig. 2:**
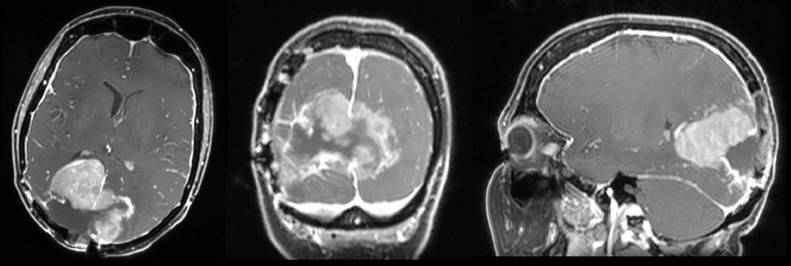
Magnetic resonance images of the brain in February 2020; axial, coronal, and sagittal image showing progression of disease of the anaplastic meningioma in both occipital lobes.

A 52-year-old woman presented to the emergency department with complaints of several days of progressive dyspnea and thoracic pain. Her medical history included a meningioma in the right occipital lobe since 2010, which was discovered after causing a homonymous hemianopia at the patient's left side. The first diagnosis was of an atypical meningioma. The tumor's driver mutation was the neurofibromatosis 2 exon 8 c.784C>T mutation. Initially, the patient was treated with surgery and adjuvant radiotherapy. In 2013, there was radiologic recurrence and she was again treated with surgery and adjuvant radiotherapy. Once more, the pathological diagnosis was atypical meningioma. The same occurred in 2015, although this time the patient received adjuvant proton therapy as well. In 2018 the follow-up MRI-scan showed possible local disease recurrence or radiation necrosis, hence dexamethasone and bevacizumab (a vascular endothelial growth factor receptor antibody) were started. The bevacizumab was stopped in the beginning 2019, because the patient suffered from polyneuropathy and she had clinically improved during treatment. However, in the second half of 2019 there was eminent disease recurrence seen on MRI in the occipital lobe (Fig. 1). No systemic or local treatment was given at that time. Meanwhile, the patient was given a ventricular-peritoneal drain, because thrombosis in the superior sagittal sinus caused an increase in intracranial pressure. Debulking of the tumor was performed and histopathological analysis showed an anaplastic meningioma with the known mutation in the neurofibromatosis gene. One month prior to presentation, a follow-up MRI-scan of the brain (Fig. 2) showed local growth of the meningioma. Since the patient suffered from visual impairment, she used 4 mg dexamethasone twice daily.

**Fig. 3:**
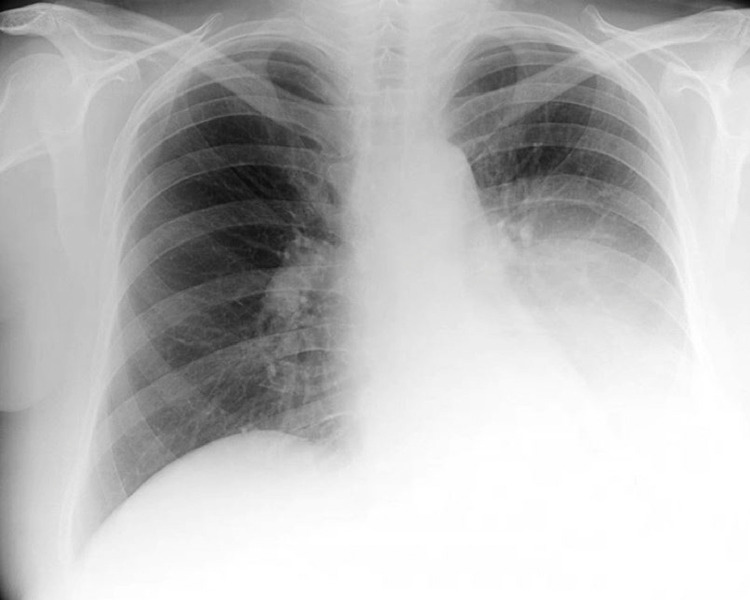
Chest X-ray at presentation; total opacification of the left lower lobe.

**Fig. 4:**
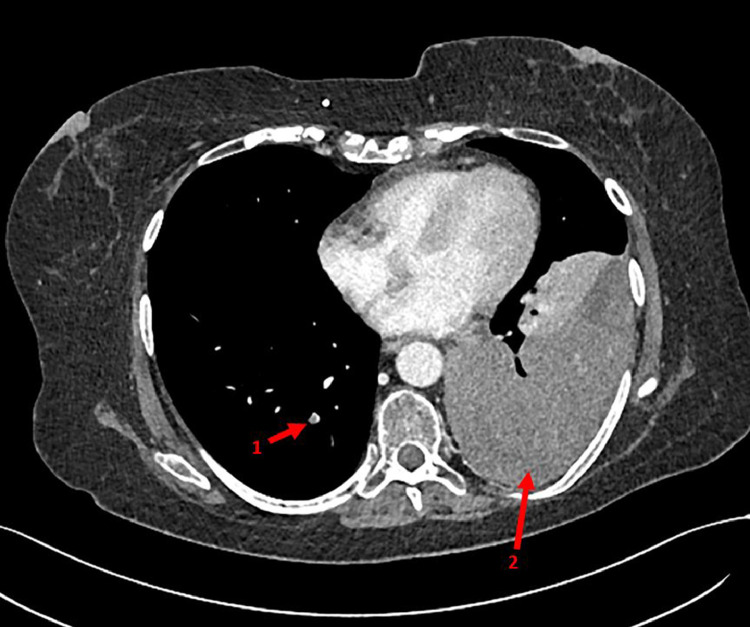
Chest computed tomography pulmonary angiography at presentation; central and right-sided subsegmental pulmonary embolisms (arrow 1), and a pleural tumour in the left lower lobe (arrow 2).

In the emergency department, the patient's vital signs revealed a mild hypoxia, for which 3 L/min supplemental oxygen was given to obtain an oxygen saturation of 95%. Her respiratory rate was 18/min and she had normal hemodynamic parameters. Blood results presented low infection parameters and an elevated d-dimer of 4.4 mg/L (normal <0.5 mg/L). A chest X-ray was made, which showed occurrence of opacification of the left lower lobe (Fig. 3), compared to a chest X ray 3 months prior to presentation. This finding was suggestive for unilateral pleural effusion, a lobular pneumonia, atelectasis, or an intrathoracic mass. Hence a computed tomography pulmonary angiography (Fig. 4) was made, that showed central and right-sided (sub-)segmental pulmonary embolisms (arrow 1), and a pleural mass of 12 × 9 × 15 cm in the left lower lobe (arrow 2).

The patient was admitted for 9 days in total, during which she was treated with low-molecular weight heparin for the pulmonary lung embolisms. Throughout the admission, the dyspnea and thoracic pain gradually improved. Meanwhile, a biopsy of the pleural mass was performed. Genome-wide profiling with a methylation assay clustered the tumor as malignant meningioma, herewith confirming the tumor to be a pleural metastasis of the anaplastic meningioma.

**Fig. 5:**
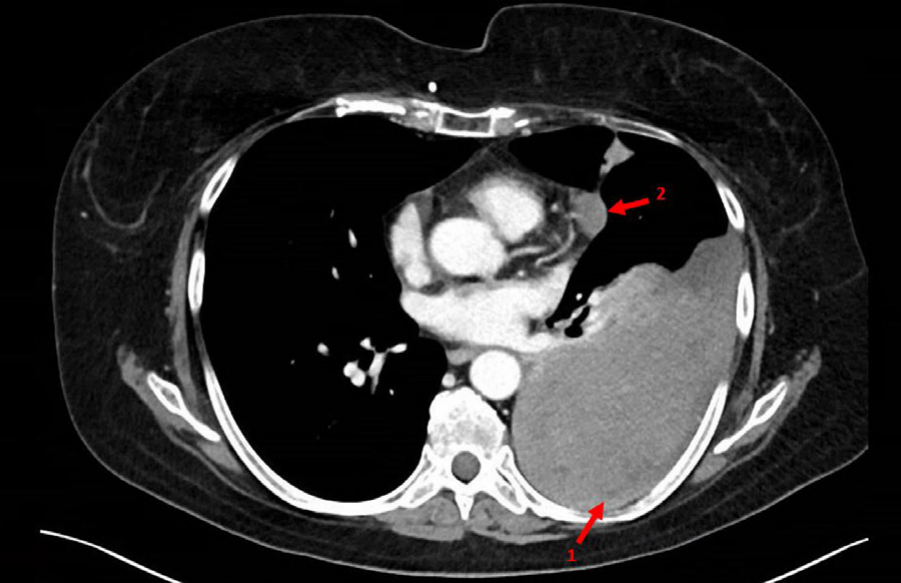
Chest computed tomography one month after presentation; growing pleural mass in the left lower lobe (arrow 1) and a new pericardial mass left (arrow 2).

In the weeks after discharge, the patient's condition and eyesight declined. A follow-up scan showed progression of the pleural metastasis and a new pericardial mass (Fig. 5). Because of further clinical deterioration, the patient did not receive further palliative anticancer treatment and she died approximately 1 month later.

## Discussion

This case report describes the discovery of a rare pleural metastasis in a patient with recurrent anaplastic meningioma. The patient presented with common respiratory complains and an elevated d-dimer. The computed tomography pulmonary angiography showed multiple pulmonary embolisms, but also a large pleural mass. This pleural tumor was pathologically diagnosed as a metastasis of the patient's known anaplastic meningioma. Extracranial metastases from a meningioma are extremely uncommon (≤ 0.1%-0.2% of cases) and involve mostly pulmonary metastases [8], [9], [10]. The size and presentation of the metastasis in this case are furthermore remarkable. Characteristic for pleural metastases is the rounded angle between the tumour and the lung parenchyma.

The patient's disease history is typical for anaplastic meningioma; after multiple local recurrences of a lower grade (in this case atypical) meningioma, it differentiates to a pathological anaplastic meningioma and gains the potency to metastasize [11]. At the time discovery in the emergency department, the primary tumor could still be from a different origin. In this case, the conclusive diagnosis could be based on specific genetic testing of the tumor and was positive for meningioma. The pathologic confirmation is essential for determination of possible treatment options; if this tumor was the metastasis of another primary tumor, more specific-and registered-therapy may have been given. This hence attributes valuable information to a patient's prognosis, because metastatic disease of anaplastic meningioma is an infaust diagnosis [11].

## Patient consent statement

The late husband of the patient was informed and gave informed consent for the usage of all necessary images and medical information for this case report.
